# The positive effect of coexisting ecosystem engineers: a unique seaweed-mussel association provides refuge for native mud crabs against a non-indigenous predator

**DOI:** 10.7717/peerj.10540

**Published:** 2020-12-21

**Authors:** Paula Tummon Flynn, Keegan McCarvill, K. Devon Lynn, Pedro A. Quijón

**Affiliations:** Department of Biology, University of Prince Edward Island, Charlottetown, PE, Canada

**Keywords:** Giant Irish moss, Blue mussel, Native mud crab, Non-indigenous green crab, Refuge, Predation

## Abstract

In marine sedimentary bottoms, mussels and macroalgae have long been recognized as important autogenic engineers that create habitat and modify abiotic conditions. The structural complexity added by bivalves and macroalgae may also mediate intraguild predation amongst marine decapod crustaceans. While spatial distributions of these ecosystem engineers frequently overlap, there is limited understanding of compounded effects when more than one engineer is present. Here we demonstrate that the coexistence of two ecosystem engineers may create habitat valuable for the survival of a small native species, the Atlantic mud crab (*Panopeus herbstii*), in the presence of the invasive green crab (*Carcinus maenas*). Using laboratory and field habitat mimics, we measured mud crab survival rates as a proxy for refuge quality. We compared the refuge provided by a unique association between shells of blue mussels (*Mytilus edulis*) and the giant strain of Irish moss (*Chondrus crispus*) to that provided by bare substrate, and by each engineer alone. These experiments revealed that the association of giant Irish moss with blue mussel shells positively and non-additively increased mud crab survival compared to the other less complex habitat mimics. In contrast, parallel experiments revealed that high habitat complexity was less important for young green crabs to survive predation from large conspecifics. These results suggest that the impact of ecosystem engineers on trophic dynamics should be considered in a broader, whole-community context encompassing multiple habitat-forming species present.

## Introduction

The positive effects of autogenic ecosystem engineers, i.e., species which create a new structural state in the environment (Jones, Lawton & Shachak, 1994; Jones et al., 2010) have gained broad recognition. These species modify abiotic conditions and resources by creating or altering habitat (Stachowicz, 2001; Ellison et al., 2005), thereby enhancing local diversity and function (Idjadi & Edmunds, 2006; Hughes, 2010). This is particularly evident in marine sedimentary bottoms, traditionally described as uniform and unstructured (see Byers & Grabowski, 2014). In these systems autogenic engineers such as seaweeds, mussels, and oysters act as foundation species, forming scattered clumps or large dense beds that provide a highly structured habitat not available in the surrounding sediments (Bouma et al., 2009; Angelini et al., 2011). These complex habitats are often critical to the early life stages of transient or resident species (Borthagaray & Carranza, 2007; Kovalenko, Thomaz & Warfe, 2012; Waser, Splinter & Van der Meer, 2015), and mobile prey seeking refuge from predators (Hughes & Grabowski, 2006; Malyshev et al., 2020). Multiple habitat-forming species often co-occur, but most studies focusing on their role have considered the effects of only a single species. This potentially underestimates the importance of these associations (Angelini et al., 2011; Gutiérrez, Bagur & Palomo, 2019) which can drive variation in biodiversity by compounding engineering effects, often sequentially through facilitation cascades (Thomsen et al., 2010; Passarelli et al., 2014; Angelini & Silliman, 2014).

The importance of ecosystem engineers in mediating predator–prey interactions has been demonstrated in coastal systems (Heck & Crowder, 1991; Grabowski, 2004; Hereu et al., 2005). For instance, several studies have compared the refuge provided by different species of bivalves or macroalgae in sedimentary bottoms (e.g., Fernández, Iribame & Armstrong, 1993; Hernández Cordero & Seitz, 2014; Waser, Splinter & Van der Meer, 2015; Gehrels et al., 2017; Malyshev et al., 2020). A few of these studies have included treatments with a mixture of two or more ecosystem engineers (e.g., Stoner, Ottmar & Haines, 2010; mussel shells and alga together) to represent habitats of higher complexity. However, little effort has been made to determine whether interactions between multiple engineers have redundant, additive, or non-additive effects on predator–prey interactions. For example, mussel beds provide hard substrata for the attachment of sessile organisms, including macroalgae, which can act as secondary engineers and provide further three-dimensional structure. This raises the question of whether the concurrent, combined effects of these species are similar to the effects provided by each of them when occurring individually. We address this question by examining a unique seaweed-mussel association that provides refuge to a native crab species in Atlantic Canadian sedimentary bottoms.

The giant Irish moss, a unique strain of the common red algae *Chondrus crispus* colloquially named after its unusually broad fronds (Sharp et al., 2010), is found solely in Basin Head, a shallow lagoon in eastern Prince Edward Island, southern Gulf of St. Lawrence. Unlike typical Irish moss which occupies outer coast habitat, giant Irish moss reproduces exclusively through fragmentation and lacks a holdfast. Its presence and survival therefore relies on an association with blue mussels (*Mytilus edulis*) that snag drifting fragments and anchor them in the shallow subtidal through byssal thread attachment (Tummon Flynn et al., 2018; Tummon Flynn et al., 2019). The giant Irish moss population has declined precipitously since 2000 (Sharp et al., 2003; Tummon Flynn et al., 2019), most likely as a result of cumulative anthropogenic environmental factors contributing to a general decline in habitat (Sharp et al., 2010; Tummn Flynn et al., 2020, unpublished data).

The loss of the giant Irish moss - blue mussel habitat may represent a threat to native species like the Atlantic mud crab (*Panopeus herbstii*). This small crab (up to ∼55 mm in carapace width (CW) is an important mid-trophic level generalist consumer in shallow water habitats (Silliman et al., 2004). Its diet consists primarily of small bivalves, barnacles, and molluscs, but can also include crustaceans, annelids, fish, macroalgae, and *Spartina* (Stachowicz & Hay, 1999; Silliman et al., 2004; Griffen & Mosblack, 2011; Whitefleet-Smith & Harding, 2014). The mud crab uses bivalve habitat in the intertidal and shallow subtidal to hide from large mobile predators (Dittel, Epifanio & Natunewicz, 1996; Grabowski, 2004; Hill & Weissburg, 2013) and is frequently found among oyster reefs, blue mussel beds and seaweed stands in the area.

One important predator of mud crabs is the non-indigenous European green crab (*Carcinus maenas*) (Gehrels et al., 2016), now broadly distributed in Atlantic Canada (Poirier et al., 2017a), and notorious for disrupting shellfish, sediments, and habitats (e.g., Cohen, Carlton & Fountain, 1995; Garbary et al., 2014), including the seaweed-mussel associations studied here (Tummon Flynn et al., 2019). This mid-size crab (up to ∼90 mm CW) preys on native crustaceans and competes with them for resources and habitat (Rossong et al., 2011; Wong & Dowd, 2014; Gehrels et al., 2016). While the green crab can be found in a wide range of habitats, they are particularly abundant in sheltered soft-bottomed habitats where mud crabs are also common in Atlantic Canada (Gehrels et al., 2016). Young green crabs use the same low intertidal and shallow subtidal beds of bivalves, algae, and seagrasses as nursery habitat and adult green crabs move on- and offshore with the tide (Hunter & Naylor 1993). Green crabs have become established in the shallow arm of the Basin Head lagoon where giant Irish moss and associated mussels were formerly abundant (Tummon Flynn et al., 2019).

Although the rareness of this unique seaweed was the principal reason for the designation of Basin Head as a Marine Protected Area (MPA) in 2005 (Sharp et al., 2010), very little is known about its role in shaping the communities present or the ecosystem-level consequences which may occur with its degradation. An understanding of how this habitat modifies interactions between species, such as predation and competition, is valuable for the effective management of the MPA. It is unclear if the intimate association between mussels and giant Irish moss offers native mud crabs a refuge that differs from the ones provided by blue mussels or Irish moss alone, or even bare sediments (e.g., MacDonald et al., 2014). Hence, this study assessed differences in refuge quality using an *a priori* null hypothesis: for native mud crabs, refuge quality does not differ across increasingly structured habitats. This hypothesis was tested in the laboratory and the field using large green crabs as predators and mud crab survival rates as proxies for prey refuge quality. Juvenile green crab survival was also measured in the same settings to assess whether habitat complexity drives the invasive crab’s survival in a similar way to that of the native mud crab.

## Methods

### Crab collection and holding

**Figure 1:**
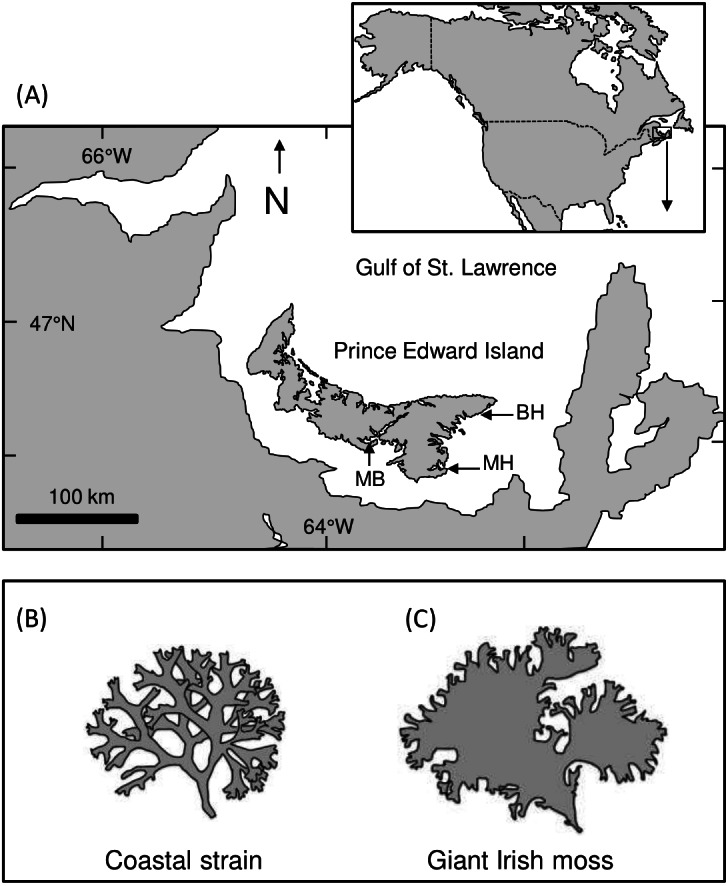
Map of Prince Edward Island and study areas. (A) Map showing Prince Edward Island (PEI) within the southern Gulf of St. Lawrence, Atlantic Canada, and the approximate location of Meadowbank (MB), Basin Head (BH), and Murray Harbour (MH). (B and C) Illustrations showing the difference between coastal and giant Irish moss strains

Large green crabs (predators; 60–80 mm shell length, SL) were collected with foldable Fukui traps (60 × 45 × 20 cm high, with a 40 cm opening at each end) deployed in Basin Head’s Northeast Arm (46°23′15′N, 62°06′19′W; Fig. 1). In parallel, mud crabs and small green crabs (prey; 25–30 mm SL and 25–35 mm SL, respectively) were collected with minnow traps (22.9 cm diameter at center, 41.9 cm long with a 2.54 cm diameter opening) deployed in Basin Head and Murray Harbour (46°03′12′N, 62°31′45′W; small green crabs) and in the West River estuarine system near Meadowbank (46°11′12′N, 63°14′40′W; mud crabs; Fig. 1A), where both species are known to occur and spatially overlap (Gehrels et al., 2016). All traps were baited with Atlantic mackerel (*Scomber scombrus*) and placed in shallow subtidal waters for 24 h. After collection, crabs were held in multiple glass tanks (21.6 cm × 41 cm × 25 cm high) for up to 1 wk and fed with mackerel every 2–3 d before their use in an experiment. Tanks used for holding crabs and for running laboratory trials (see below) were filled with prepared seawater (Instant Ocean and groundwater, ∼25 ppt, 18−20 °C) and aerated with an airstone. Collection and handling of organisms was all done with approved annual permits from the Department of Fisheries and Oceans Canada (SG-RHQ-15-034, SG-RHQ-16-026 and SG-RHQ-17-053).

### Habitat mimic preparation

Four distinct habitat mimics were prepared for laboratory and field trials to represent habitats of increasing physical complexity: bare substrate, blue mussel shells, giant Irish moss, and a combination of blue mussel shells and giant Irish moss. Bare substrate treatments lacked shells and seaweeds in laboratory and in field trials, where they consisted of no substrate (glass tanks with water only) in the laboratory and of bare sandy sediments in the field. Sediments in the field site are muddy so it was not practical to add a layer in the laboratory tanks without a drastic rise in turbidity that would preclude observations. In addition, previous studies with these species (Gehrels et al., 2016; Gehrels et al., 2017; Malyshev et al., 2020) have shown that the addition of sediments does not significantly change predation rates by large green crabs compared to bare glass bottoms. Blue mussel habitats were mimicked with a three cm layer of empty (mostly disarticulated) shells (∼3.5–4.5 cm SL) collected near Meadowbank and washed prior to the experiments. The empty shells were placed in the middle of laboratory tanks and field cages and arranged to create discrete clumps with diameters similar to those recorded on the seabed in Basin Head (Sharp et al., 2010; I Novaczek, Department of Fisheries and Oceans Canada, pers. comm., 2018) and to take up approximately 20–30% of the area, leaving a perimeter of bare substrate around the clump. In the laboratory tanks, clumps were ∼15 cm in diameter, while in the larger field cages (see dimensions below), clumps were ∼30 cm in diameter. Because of limited availability and protected status of the giant Irish moss growing in Basin Head, giant Irish moss habitats were created with fronds obtained from the National Research Council of Canada facility in Sandy Cove, Nova Scotia, where an original stock from Basin Head has been maintained in tank culture to preserve this distinctive strain (Figs. 1B, 1C). The Irish moss was obtained and shipped from Sandy Cove in May 2018 and hung in mussel socking (sleeves of plastic mesh used in aquaculture) on floating lines in Basin Head for at least one month before its use.

Mimics of giant Irish moss clumps were created by sewing together individual fronds with fine twine (see Tummon Flynn et al., 2019) and then tying them to either a suction cup or a tent peg to hold them to the bottom of a tank or the seafloor, respectively. Giant Irish moss clumps were ∼15 cm in diameter (∼50 g wet weight after dewatering for 30 s in a salad spinner) and ∼30 cm in diameter (∼300 g), for laboratory and field trials, respectively. Mimics of giant Irish moss and blue mussel shells combined were prepared in a similar way, keeping the overall size of the clumps the same (15 and 30 cm diameter), and placing the sewn giant Irish moss in the center of the pile of blue mussel shells. After the 24 h trials, all three habitat mimics (mussel shells, giant Irish moss, and giant Irish moss + blue mussels) consistently held their clump integrity in shape and size, both in the laboratory and the field.

### Experimental procedure

The sides and top of tanks used for laboratory trials were covered to darken the environment and to reduce distracting visual stimuli (see Pickering & Quijón, 2011; Pickering et al., 2017). Prior to the beginning of the experiments, the large green crabs (predators) were starved for 48 h to standardize hunger levels (e.g., Mascaró & Seed, 2001). Only intact male crabs were used to reduce variability associated with sex or injury (e.g., Smallegange & Van der Meer, 2003; Tummon Flynn et al., 2015) and each predator was used only once to avoid the risk of bias due to learning (e.g., Cunningham & Hughes, 1984). Five prey (either mud crabs or small green crabs) were placed in each tank for 30 min to acclimate before a single predator (large green crab) was introduced. Feeding trials ran for 24 h, after which the experiment was terminated and prey survival (number of unharmed small crabs) was recorded. The position of any surviving prey within the available habitat was also recorded. All the trials were conducted during summer months (July-August).

Due to restrictions stemming from the status of Basin Head as a Marine Protected Area, field experiments were conducted in Meadowbank. Similar to Basin Head, the study site was dominated by muddy and sandy sediments characterized by scattered eelgrass beds, mussel and oyster beds, epibiont macroalgae populations attached to bivalve shells, and fringing salt marsh vegetation. Crabs were placed in field cages (50 cm × 50 cm × 60 cm high) built with plastic coated wire with 1 × 1 cm mesh and placed every ∼10 m along the upper subtidal. The cages had open bottoms that allowed us to insert them 5–10 cm into the sediment to prevent crab escape. Cages were placed just below the intertidal zone, where both crab species have been trapped, so that crabs and habitats remained submerged under at least 50 cm of water throughout the tidal cycle. This positioning prevented desiccation stress and ensured large crabs would be active and hunting effectively in order to detect potential differences in survival rates. After 24 h, prey survival was checked by counting live crabs and sieving (one mm mesh) the top 2–3 cm of sediment to collect and count carapace remains. Trials where a crab was unaccounted for were not included in further analyses. If little or no mortality was recorded after 24 h, the trials were left running and checked again at 48 h. In those cases, survival rates were calculated as survival over two days and then expressed as crab survival per day (no differences were found between survival rates measured during the first and the second day). Trials with each type of habitat mimic in the laboratory or the field were replicated between 8 and 16 times (see Table 1).

**Table 1:** Habitat mimics, replicates, mean and median survival rates. Habitat mimics, number of replicates (*n*), mean (±SE), median, and mean rank survival for two species of prey (mud crabs and small green crabs) exposed to predation by large green crabs in laboratory and field conditions.

Setting	Prey	Habitat	*n*	Mean survival ( ± SE)	Median survival	Mean rank
Laboratory	Mud crabs	Bare sand	15	0.53 (0.29)	0	19.03
		Mussel shells	12	1.67 (0.31)	1	36.04
		Irish moss	12	1.33 (0.45)	0.5	30.04
		Irish moss + shells	12	4.25 (0.37)	5	75.83
	Green crabs	Bare sand	15	2.07 (0.46)	2	41.80
		Mussels	13	3.54 (0.37)	4	62.42
		Irish moss	12	4.42 (0.26)	5	77.38
		Irish moss + shells	12	4.75 (0.13)	5	83.38
Field	Mud crabs	Bare sand	16	0.75 (0.30)	0	19.09
		Mussel shells	9	0.56 (0.24)	0	17.67
		Irish moss	16	1.94 (0.40)	2	32.88
		Irish moss + shells	12	3.33 (0.45)	3.5	53.75
	Green crabs	Bare sand	14	4.64 (0.15)	5	73.64
		Mussel shells	8	4.06 (0.26)	4.25	58.38
		Irish moss	12	4.63 (0.11)	4.5	70.83
		Irish moss + shells	12	4.88 (0.09)	5	80.54

### Data analysis

Normality and heterogeneity of data were checked using the Anderson Darling test and Levene’s test, respectively. Due to repeated violations of normality assumptions, a rank two-way ANOVA model was used to examine crab survival (response variable) associated with two main factors: prey species (small green crab vs. mud crab), habitat (bare sand vs. mussel shells vs. giant Irish moss vs. Irish moss + mussel shells), and their interaction. When the analyses showed significant interactions, a Kruskal–Wallis one-way analysis of variance on ranks was carried out for each factor separately in each level from the other factor, followed by Dunn’s method for multiple pairwise comparisons.

## Results

For both laboratory and field experiments, prey species, habitat type, and their interaction were significant (rank two-way ANOVAs, *p* < 0.05; Tables 1 and 2).

**Figure 2:**
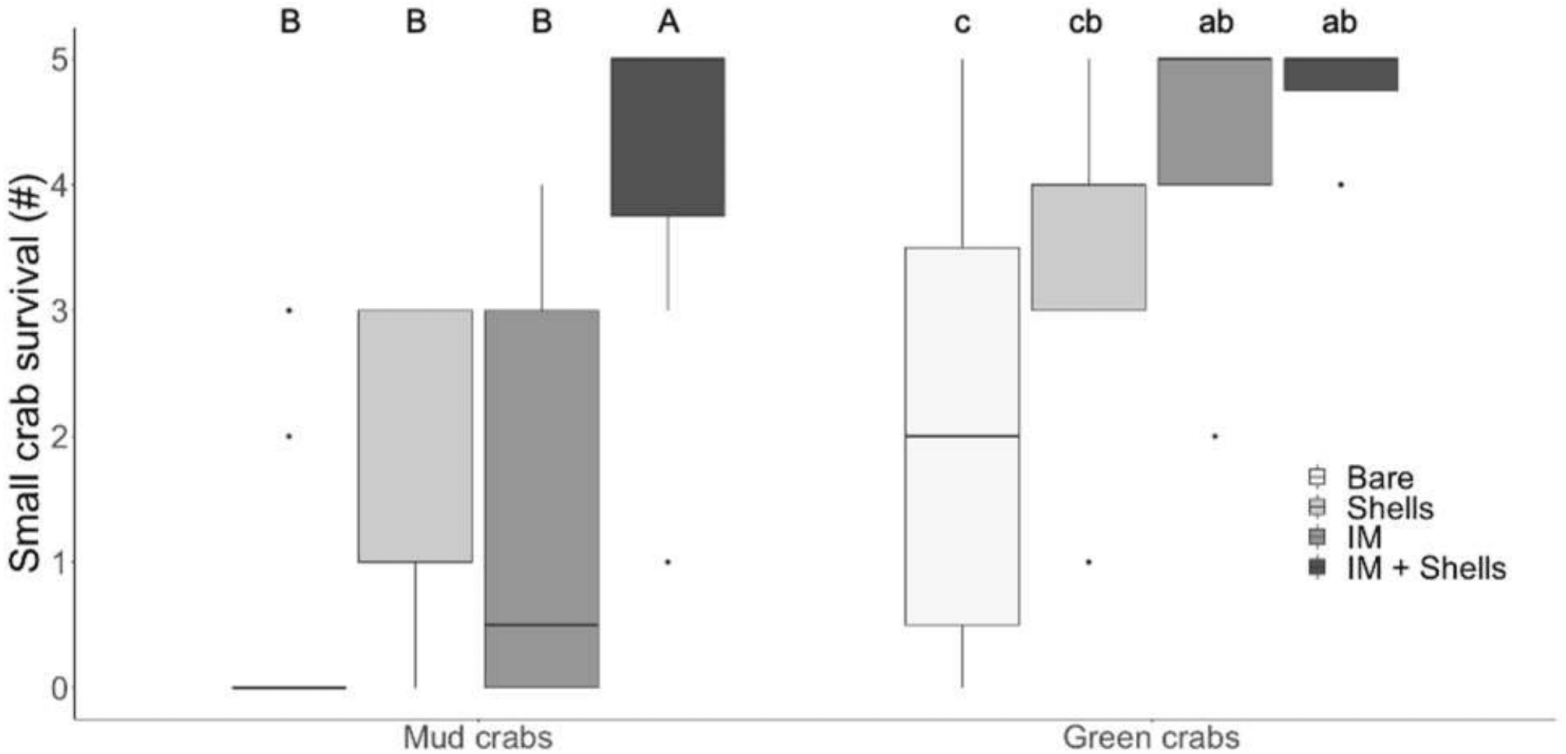
Crab survival rates in the laboratory. Mud crab and juvenile green crab survival in four habitats in the laboratory. Letters identify significant differences based on Dunn’s tests (*p* < 0.05). Different letter cases identify separate simple effects analyses. Bare, Bare sediments; Shells, Blue mussel shells; IM, Giant Irish moss. Boxplots, box indicates the 25th and 75th quartiles, horizontal line represents the median, whiskers represent the minimum and maximum, and points represent outliers that are outside 1.5 times the interquartile range.

In the laboratory trials, simple effects analysis indicated mud crab survival was affected by habitat type (Kruskal–Wallis , *H*_3_ = 27.48, *p* < 0.001). The most complex habitat mimic (Irish moss + mussel shells) was the only one that provided significantly more protection from green crab predation than bare sediment, and mud crabs experienced significantly greater survival in it than in either mussel shells or Irish moss alone (Dunn’s test; Fig. 2A). This higher survival rate was non-additive, with a median survival of 5, compared to median survivals of 0.5 and 1 for mussel shells and Irish moss alone, respectively (Tables 1 and 2; Fig. 2). For small green crab prey, survival was also affected by the habitat type (Kruskal–Wallis, *H*_3_ = 20.98, *p* < 0.001). The Irish moss + mussel shells habitat and mussel shells alone habitat both provided greater protection than bare sediment (Tables 1 and 2; Fig. 2). Observations of surviving prey revealed that in the seaweed-mussel clumps, most mud crabs (90%) sheltered among the mussel shells. In contrast, surviving small green crabs were usually found within the Irish moss or moving indiscriminately around the tank, among fronds, shells and above or below the clumps.

**Table 2:** ANOVA results for laboratory and field trials. Results of ranked two-way ANOVAs examining the effects of prey species, habitat type, and their interaction on the survival of mud crabs and small green crabs. Significant *p*-values are bolded. Results of pairwise comparisons using Dunn’s methods are illustrated in Figs. 2 and 3.

Setting	Main factor	DF	MS	*P* value
Laboratory	Prey species	1	17,260.2	**<0.001**
	Habitat type	3	10,840.8	**<0.001**
	Prey × Habitat	3	1,617.8	**0.004**
	Error	95	344.6	
Field	Prey species	1	37,510.5	**<0.001**
	Habitat type	3	3,247.6	**<0.001**
	Habitat × prey	3	881.4	**0.020**
	Error	91	256.7	

**Figure 3:**
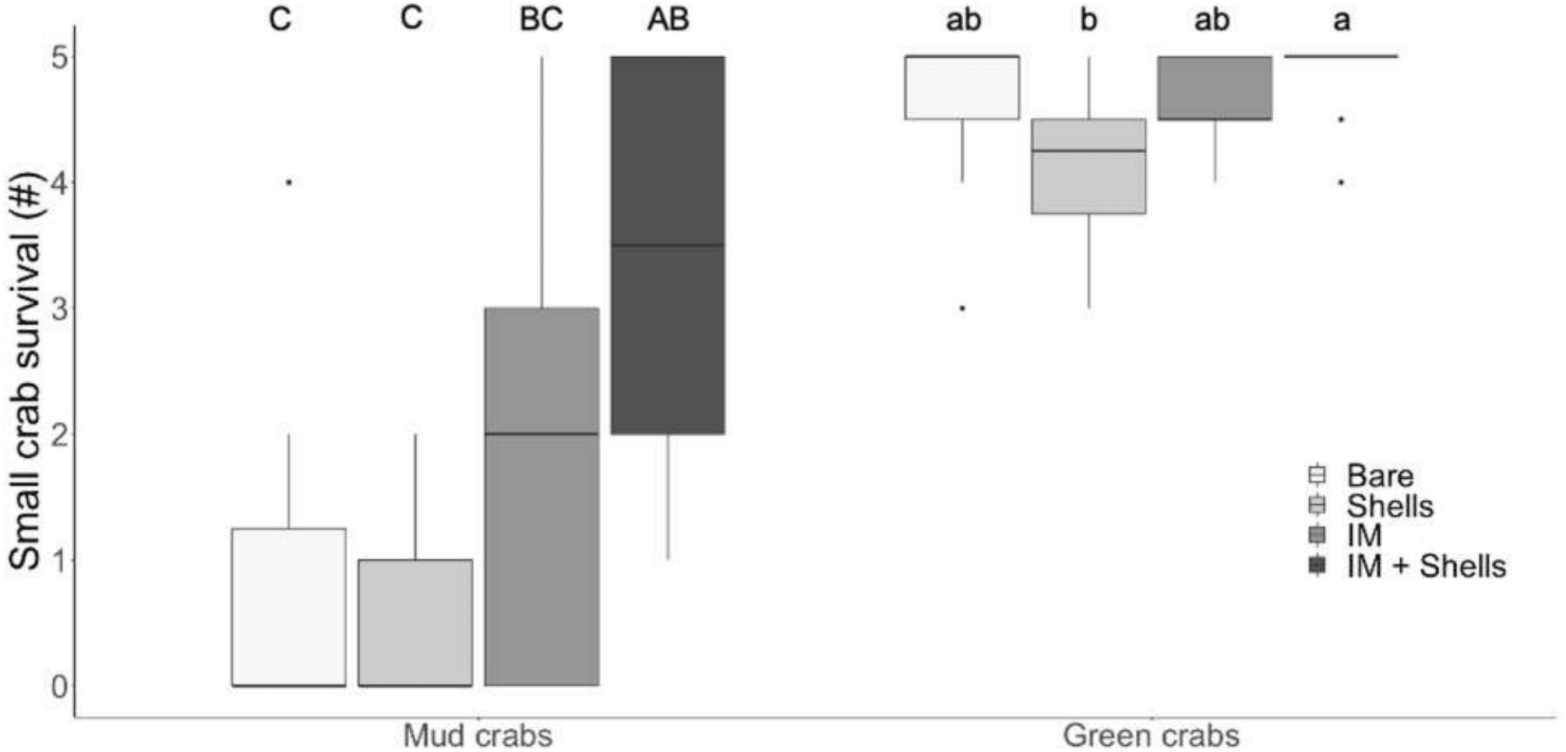
Crab survival rates in the field. Mud crab and juvenile green crab survival in four habitats in the field. Letters identify significant differences based on Dunn’s tests (*p* < 0.05). Different letter cases identify separate simple effects analyses. Bare, Bare sediments; Shells, Blue mussel shells; IM, Giant Irish moss. Boxplots, box indicates the 25th and 75th quartiles, horizontal line represents the median, whiskers represent the minimum and maximum, and points represent outliers that are outside 1.5 times the interquartile range.

In the field trials, mud crab survival was significantly affected by habitat type (Tables 1 and 2; Fig. 3; Kruskal–Wallis, *H* = 19.85, *p* < 0.001). The only habitat that provided significantly more protection than bare sediments was the most complex habitat, i.e., Irish moss + mussel shells. Again, the increase in mud crab survival was non-additively positive when the two habitat mimics were combined, with a median survival rate of 3.5 compared to medians of 0 and 2 in mussel shells alone and Irish moss alone, respectively. For small green crab prey, survival rates were high across all habitats, ranging between 4.25 and 5 crabs d^−1^; however survival was still affected by habitat type (Tables 1 and 2; Fig. 3; Kruskal–Wallis , *H*_3_ = 10.91, *p* < 0.05) due to a significant difference between mussel shells (median of 4.25) and Irish moss + mussel shells (median of 5) (Fig. 3).

## Discussion

This study supports the idea that predator–prey interactions in soft-bottom marine environments can be shaped by the presence of autogenic ecosystem engineers (Hughes & Grabowski, 2006; Gehrels et al., 2016). It also provides evidence that the association of two of these species can have compounding engineering effects on trophic interactions. Our results demonstrate that native mud crabs find refuge from predation by invasive green crabs in a habitat created by the association of giant Irish moss with blue mussel shells. In both laboratory and field trials, this particular habitat offered superior refuge quality for mud crabs compared to habitats with a single ecosystem engineer (giant Irish moss or blue mussel shells) alone, neither of which improved mud crab survival significantly compared to bare sediments. We therefore reject our null hypothesis and highlight the non-additive positive effects provided by the co-existence of these autogenic engineers (Angelini et al., 2011; Gutiérrez, Bagur & Palomo, 2019). These results add to other studies that demonstrate the effects of two engineers together are not always possible to predict from individual effects when they impact the same habitat or resource (Boye & Fong, 2005; Passarelli et al., 2014).

While we did not dissect the mechanisms that underlie higher mud crab survival in Irish moss—mussel shell clumps, prior studies have linked enhanced prey survival to greater habitat complexity (Hughes & Grabowski, 2006; Waser, Splinter & Van der Meer, 2015; Schmitz et al., 2017). A rise in complexity can reduce predator–prey encounters or increase predator handling time, resulting in a reduction in predator foraging efficiency (Lima & Dill, 1990). Bivalve shell substrates create crevices and interstitial spaces and have been shown to provide hideouts from predators (Grabowski, 2004; Gehrels et al., 2016). The foliose structure of red algae (e.g., *Laurencia* spp. and *Gracilaria* spp.) has also been shown to add physical and visual background complexity, both of which favor prey concealment (Heck Jr & Orth, 1980; Orth, Heck & Van Montfrans, 1984) and reduce predator capture rates (Dionne & Folt, 1991). The attachment to and entanglement of the broad fronds of giant Irish moss with blue mussels provides further 3-dimensional structure that was particularly beneficial to mud crabs, likely throughdecreased detectability and accessibility by predators. Similar associations between blue mussels and macroalgae have been described where algal epibionts have physically modified the habitat, increasing structural complexity and diversity, and changed environmental conditions under algal thalli in mussel beds (e.g., *Fucus vesiculosus*, Albrecht & Reise, 1994; *Porphyra sp.,* Gutiérrez, Bagur & Palomo, 2019). Our laboratory observations suggest that mud crabs preferentially shelter amongst and underneath mussel shells embedded in the clumps rather than within the algal fronds, which is not surprising as xanthid mud crabs are known to hide under shells and rocks (Kneib & Weeks, 1990; Silliman et al., 2004). The overhead coverage and added visual complexity provided by the Irish moss likely enhanced the benefits of this behaviour.

Green crabs commonly cannibalize younger stage conspecifics (Moksnes, Pihl & Van Montirans, 1998; Gehrels et al., 2017), and in the absence of alternative prey, this may account for some of the natural mortality affecting this species (see Moksnes, Pihl & Van Montirans, 1998; Moksnes, 2004). However, in our study cannibalism rates were generally lower than predation on native mud crabs in most habitats and juvenile green crab survival was not as dependent on habitat complexity. Although laboratory experiments showed that some complex habitats (mussel shells and Irish moss + mussel shells) increased small green crab survival compared to bare substrate, a similar pattern was not found in field experiments. In the larger field cages, where small green crabs had more space to escape from predators as well as the option of digging or burrowing into the sand, survival was high in most habitat mimics. The lack of consistency in green crab survival rates in the different habitat mimics reflects either a stronger ability of juvenile green crabs to escape predation using a variety of habitats (algae, bivalve shells or a combination of them; Thiel & Dernedde, 1994; Amaral et al., 2009), their ability to effectively evade capture (run away) regardless of habitat availability, or a combination of both. Laboratory observations of surviving juvenile green crabs using both seaweeds and mussel shells for shelter when they were simultaneously available, as well as observations of predator evasion by moving around the tanks, suggest a combination of both strategies. In comparison, native mud crabs were less mobile and more reliant on the added physical structure provided by the association between giant Irish moss and mussel shells.

The use of empty mussel shells cannot fully reproduce the complex, hierarchical structure of natural mussel matrices which includes shell debris with fragmented and whole shells, byssal threads, epibionts, and live mussels producing and depositing organic material within the bed (see Commito & Rusignuolo, 2000; Crawford, Commito & Borowik, 2006; Tummon Flynn et al., 2019). While we acknowledge that the use of shell instead of live mussels is a limitation, the preservation of the experimental clump’s integrity over the course of these short-term experiments, with shells as a substrate and giant Irish moss fronds anchored among them and extending into the water column, appears to have reasonably mimicked what mussel byssal threads do in nature. Giant Irish moss grows on blue mussels and, lacking holdfasts, it is it is attached to the substratum by byssal threads, which the mussels produce fairly quickly to anchor new fronds (formation of new algal-mussel clumps in laboratory conditions within 48 h; Tummon Flynn et al., 2019). This association, as with other seaweed-bivalve associations (e.g., *Fucus vesiculosus* and blue mussels in the Wadden Sea; Albrecht, 1998), first and foremost generates distinct clumps and biogenic reefs that control species biodiversity through physical structural properties, but in the long-term can also change the characteristics of the sediment and provide critical functional services (Albrecht & Reise, 1994; Bouma et al., 2009). Increased habitat complexity may also attract predators (Thiel & Dernedde, 1994; Langellotto & Denno, 2006) and chemical cues released from living mussels (Wilson & Weissburg, 2012) could increase predator pressure on small crabs. The study of the role of those chemical cues was beyond the scope of this study but represents an interesting venue for future studies. Alternatively, the presence of living mussels below refuge sizes (Jubb, Hughes & Rheinallt, 1983) could decrease predation pressure on crabs by providing alternative prey (see Poirier et al., 2017b). Indeed, in laboratory and field trials green crabs have been shown to interact heavily with giant Irish moss—blue mussel clumps and to successfully detach small blue mussels from the clump prior to consumption (Tummon Flynn et al., 2019). Although these potential effects were not accounted for here, this does not alter the validity of the habitat differences found: a good representation of the structural integrity of the natural clumps (the main role played by byssal threads) was maintained for the duration of the trials using mussel shells and sewn giant Irish moss as habitat mimics.

The primary conclusion of this study is that the coexistence of habitat-forming engineering species can create highly valuable refuge habitat and potentially decrease the vulnerability of native species to invasive predators. Hence, measures to protect and conserve giant Irish moss and blue mussels most likely benefit mud crabs in the area as well, as the seaweed-mussel associations reduce mortality by green crab predation. While giant Irish moss has been the subject of regulatory protection by a Marine Protected Area since 2005 due to its rareness (Sharp et al., 2010; Tummon Flynn et al., 2018), as it is generally stated for e.g., umbrella and charismatic species (*sensu* Caro, 2010), the protection of this strain also serves the conservation needs of other species as a foundation species (Scrosati, 2016) and as a co-engineer (e.g., Gutiérrez, Bagur & Palomo, 2019) with blue mussels. Exploring such positive (facilitative) roles is important to fully understand the interactions between a diverse array of organisms and this seaweed-mussel association, and is also relevant from a pragmatic point of view: Protection measures for threatened or unique species may be difficult to sustain in the long-term (Camacho et al., 2010), so identifying potential benefits for other native species can provide additional arguments for ongoing conservation efforts. The fact that small green crabs did not show the same reliance on high habitat complexity to avoid conspecific predators indicates that depletions in foundation species (e.g., the >99% loss of giant Irish moss biomass in Basin Head since 2000; Tummon Flynn et al., 2019) can disproportionately affect native species.

The success of conservation efforts and the recovery of the giant Irish moss—blue mussel population will dictate further studies on this unique association of ecosystem engineers. These studies should focus on the facilitation roles of these foundation species for native organisms seeking refuge in the algal-mussel clumps (e.g., peracarid amphipods; Tummon Flynn et al., 2020, unpublished data). Interpreting the full impact of this loss of habitat and the parallel green crab invasion on mud crabs will depend on the loss of other services provided by this biogenic habitat and patterns of coexistence between the native and invasive crabs within the facilitation cascade (Altieri & Irving, 2017). These patterns have likely changed during the decline as large dense clumps gradually became less abundant. Habitat size and density can determine refuge quality, with many studies reporting a general decline in predator success as habitat density increases, linked to decreasing edge to interior ratios (Hovel, 2003) and decreased detectability of prey (Bartholomew, 2002). For small juvenile blue crabs, survival has been found to increase with seagrass shoot density and within large patches of *Gracilaria* (Hovel & Lipcius, 2001; Falls, 2008). Although, this relationship between habitat size and survival can be reversed when larger mobile predators, such as adult crabs, prefer to congregate in larger patches (Hovel & Lipcius, 2001) or after juvenile crabs grow above a certain size (Falls, 2008). Similarly, the potential intra-guild interactions between small crabs (native mud crabs and juvenile green crabs) sheltering within and between the clumps warrant particular attention, as habitat complexity can also influence interference competition (Grabowski & Powers, 2004) and non-consumptive effects of predators (see Belgrad & Griffen, 2016; Scherer, Garcia & Smee, 2017). The differences found here reinforce the idea that further attention should be given to the role played by prevalent assemblages of multiple engineers (Angelini et al., 2011), including widespread natural clumps, often mingling two or more ecosystem engineers (e.g., Bouma et al., 2009; Buschbaum et al., 2009). While the seaweed-bivalve association examined in this study is confined to a single location and, even prior to its decline, occupied only a fraction of the Basin Head lagoon system, macroalgal epibionts on bivalves are widely distributed in Atlantic Canada, including common Irish moss attached to blue mussels via holdfasts. Therefore, this study’s limited geographic findings may act as a model that could be reproduced in more widely distributed species of co-engineers.

##  Supplemental Information

10.7717/peerj.10540/supp-1 Supplemental Information 1 Crab survival rates in the laboratory and field.

## Additional Information and Declarations

### Field Study Permissions

The following information was supplied relating to field study approvals (i.e., approving body and any reference numbers): Field experiments and collections were approved by the Department of Fisheries and Oceans Canada - (Permits to collect for scientific purposes: SG-RHQ-15-034, SG-RHQ-16-026, SG-RHQ-17-053).

### Data Availability

The following information was supplied regarding data availability: Mud crab and green crab survival rate data are summarized in the Tables and boxplot Figures. In addition, raw data are available in a Supplemental File.
